# Supplementary material for: Modeling glioblastoma heterogeneity as a dynamic network of cell states
Source: Mol Syst Biol. 2021 Sep 16;17(9):e10105. doi: 10.15252/msb.202010105 (PMC8444284; doi:10.15252/msb.202010105)
Supplement: Supplementary file 6 — Source Data for Figure 5 [file MSB-17-e10105-s004.zip › Figure5A_sourcedata/GSEA_3017/hallmarks_stateD.GseaPreranked.1621934542601/HALLMARK_MITOTIC_SPINDLE.html]

Details for gene set HALLMARK\_MITOTIC\_SPINDLE[GSEA]

|  || Dataset | state23017 |
| Phenotype | NoPhenotypeAvailable |
| Upregulated in class | na\_neg |
| GeneSet | HALLMARK\_MITOTIC\_SPINDLE |
| Enrichment Score (ES) | -0.35653168 |
| Normalized Enrichment Score (NES) | -1.7710992 |
| Nominal p-value | 0.007322176 |
| FDR q-value | 0.07881866 |
| FWER p-Value | 0.315 |
Table: GSEA Results Summary

  

Fig 1: Enrichment plot: HALLMARK\_MITOTIC\_SPINDLE      
 Profile of the Running ES Score & Positions of GeneSet Members on the Rank Ordered List

  

| PROBE | GENE SYMBOL | GENE\_TITLE | RANK IN GENE LIST | RANK METRIC SCORE | RUNNING ES | CORE ENRICHMENT || 1 | TRIO |  |  | 76 | 0.314 | -0.0483 | No |
| 2 | MARCKS |  |  | 96 | 0.295 | -0.0522 | No |
| 3 | BIRC5 |  |  | 130 | 0.271 | -0.0681 | No |
| 4 | ARHGAP10 |  |  | 176 | -0.252 | -0.0942 | No |
| 5 | RFC1 |  |  | 190 | -0.253 | -0.0950 | No |
| 6 | NF1 |  |  | 254 | -0.260 | -0.1350 | No |
| 7 | SASS6 |  |  | 261 | -0.260 | -0.1299 | No |
| 8 | SORBS2 |  |  | 318 | -0.265 | -0.1643 | No |
| 9 | KNTC1 |  |  | 329 | -0.267 | -0.1621 | No |
| 10 | FLNA |  |  | 338 | -0.267 | -0.1583 | No |
| 11 | CDC42BPA |  |  | 415 | -0.275 | -0.2081 | No |
| 12 | CEP57 |  |  | 418 | -0.275 | -0.1993 | No |
| 13 | TUBGCP5 |  |  | 443 | -0.279 | -0.2077 | No |
| 14 | ECT2 |  |  | 481 | -0.283 | -0.2263 | No |
| 15 | PCNT |  |  | 485 | -0.283 | -0.2180 | No |
| 16 | SPTBN1 |  |  | 511 | -0.286 | -0.2270 | No |
| 17 | TUBGCP3 |  |  | 512 | -0.287 | -0.2161 | No |
| 18 | ACTN4 |  |  | 604 | -0.301 | -0.2768 | No |
| 19 | PRC1 |  |  | 645 | -0.310 | -0.2968 | No |
| 20 | CDK5RAP2 |  |  | 651 | -0.311 | -0.2890 | No |
| 21 | NUMA1 |  |  | 653 | -0.311 | -0.2780 | No |
| 22 | MID1 |  |  | 680 | -0.315 | -0.2866 | No |
| 23 | KIF4A |  |  | 706 | -0.320 | -0.2943 | No |
| 24 | RANBP9 |  |  | 728 | -0.324 | -0.2987 | No |
| 25 | CEP192 |  |  | 802 | -0.345 | -0.3435 | Yes |
| 26 | BRCA2 |  |  | 808 | -0.347 | -0.3343 | Yes |
| 27 | MYH9 |  |  | 820 | -0.350 | -0.3298 | Yes |
| 28 | PCM1 |  |  | 834 | -0.353 | -0.3267 | Yes |
| 29 | ARFGEF1 |  |  | 845 | -0.356 | -0.3211 | Yes |
| 30 | CENPJ |  |  | 849 | -0.357 | -0.3100 | Yes |
| 31 | NEK2 |  |  | 861 | -0.359 | -0.3051 | Yes |
| 32 | SMC1A |  |  | 885 | -0.367 | -0.3094 | Yes |
| 33 | DST |  |  | 913 | -0.379 | -0.3164 | Yes |
| 34 | INCENP |  |  | 914 | -0.379 | -0.3020 | Yes |
| 35 | PDLIM5 |  |  | 941 | -0.390 | -0.3079 | Yes |
| 36 | APC |  |  | 960 | -0.398 | -0.3071 | Yes |
| 37 | KIFAP3 |  |  | 988 | -0.409 | -0.3130 | Yes |
| 38 | NEDD9 |  |  | 993 | -0.412 | -0.3005 | Yes |
| 39 | KIF11 |  |  | 1003 | -0.416 | -0.2919 | Yes |
| 40 | SAC3D1 |  |  | 1025 | -0.429 | -0.2923 | Yes |
| 41 | TPX2 |  |  | 1034 | -0.437 | -0.2821 | Yes |
| 42 | KIF22 |  |  | 1051 | -0.446 | -0.2778 | Yes |
| 43 | PLK1 |  |  | 1082 | -0.460 | -0.2842 | Yes |
| 44 | DLG1 |  |  | 1100 | -0.469 | -0.2799 | Yes |
| 45 | RACGAP1 |  |  | 1112 | -0.479 | -0.2704 | Yes |
| 46 | TTK |  |  | 1119 | -0.487 | -0.2568 | Yes |
| 47 | ARHGAP29 |  |  | 1121 | -0.487 | -0.2391 | Yes |
| 48 | NUSAP1 |  |  | 1154 | -0.515 | -0.2449 | Yes |
| 49 | KIF2C |  |  | 1183 | -0.558 | -0.2460 | Yes |
| 50 | KIF15 |  |  | 1213 | -0.610 | -0.2458 | Yes |
| 51 | SMC4 |  |  | 1224 | -0.638 | -0.2296 | Yes |
| 52 | ANLN |  |  | 1229 | -0.646 | -0.2083 | Yes |
| 53 | KIF23 |  |  | 1265 | -0.741 | -0.2080 | Yes |
| 54 | AURKA |  |  | 1271 | -0.763 | -0.1830 | Yes |
| 55 | NDC80 |  |  | 1278 | -0.810 | -0.1571 | Yes |
| 56 | PIF1 |  |  | 1299 | -0.987 | -0.1355 | Yes |
| 57 | FBXO5 |  |  | 1301 | -0.992 | -0.0987 | Yes |
| 58 | CDK1 |  |  | 1311 | -1.433 | -0.0515 | Yes |
| 59 | TOP2A |  |  | 1313 | -1.528 | 0.0055 | Yes |
Table: GSEA details [plain text format]

  

Fig 2: HALLMARK\_MITOTIC\_SPINDLE: Random ES distribution      
 Gene set null distribution of ES for **HALLMARK\_MITOTIC\_SPINDLE**
